# Deep Learning Discrimination of Colorectal Cancer using Retinal Fundus Photographs: UK Biobank Case-Control Study

**DOI:** 10.2174/0115734056494970260902110339

**Published:** 2026-09-11

**Authors:** Ivan Jovovic, Tomo Popovic, Isidora Rubezic Lukic, Dejan Babic, Natasa Popovic

**Affiliations:** 1 Faculty for Information Systems and Technologies, University of Donja Gorica, Podgorica, Montenegro; 2 Faculty of Medicine, University of Montenegro, Podgorica, Montenegro; 3 Internal Medicine Clinic, Clinical Center of Montenegro, Podgorica, Montenegro

**Keywords:** Retinal fundus images, Colorectal cancer, Deep learning, EfficientNet, UK Biobank, Medical imaging, Non-invasive biomarkers

## Abstract

**Introduction/Objective:**

Retinal fundus photography captures microvascular and neuroretinal features reflecting systemic health. While Deep Learning (DL) has enabled extraction of cardiometabolic and aging biomarkers from retinal images, its application to oncologic disease status discrimination remains largely unexplored. This study evaluated whether DL analysis of retinal fundus photographs can discriminate individuals with colorectal cancer (CRC) from those without CRC (non-CRC) in a curated UK Biobank case-control setting.

**Methods:**

This retrospective imaging study used 1,600 UK Biobank fundus photographs (800 CRC, 800 non-CRC). Images underwent grayscale conversion, intensity normalization, and resizing with minimal preprocessing. Data were split at the participant level into training, validation, and test sets (70/15/15). An ImageNet-pretrained EfficientNet-B3 convolutional neural network (CNN) was fine-tuned end-to-end for binary classification. Model performance was assessed using Area Under the Receiver Operating Characteristic Curve (AUROC). Prevalence-adjusted predictive values were estimated to approximate real-world classification performance.

**Results:**

On the independent test set, the model achieved AUROC = 0.945, accuracy = 87.08%, macro-F1 = 87.07%, sensitivity = 90.00%, specificity = 84.17%, and precision (PPV in the balanced test set) = 85.04%. When recalibrated to population prevalence, PPV decreased to 2.8% at 0.5% prevalence (and 6.5% at 1.2% prevalence), whereas NPV remained high (99.94% and 99.86%, respectively).

**Discussion:**

High discrimination achieved from retinal images alone supports the presence of CRC-associated retinal signatures. A covariates-only baseline using age and vascular/heart disease showed only modest discrimination (AUROC ≈ 0.63), making measured confounders an unlikely sole explanation for model performance, although the cross-sectional case-control design and possible residual confounding preclude causal or pre-diagnostic claims. Together with the low prevalence-adjusted PPV and high NPV, these findings position retinal analysis as a potential triage or rule-out tool rather than a stand-alone diagnostic classifier.

**Conclusion:**

DL analysis of retinal fundus photographs can discriminate CRC status with high accuracy in a case-control setting, supporting the presence of CRC-associated retinal signatures. Prevalence-adjusted projections indicate limited stand-alone screening utility, positioning retinal AI analysis primarily as a potential triage or risk-enrichment tool pending longitudinal validation.

## 1 INTRODUCTION

Colorectal Cancer (CRC) is a major cause of cancer-related morbidity and mortality, with prognosis strongly dependent on stage at diagnosis [1]. While established screening methods are effective, their reach is limited by invasiveness, cost, and adherence [2, 3]. This has sustained interest in identifying complementary, non-invasive screening methods that could support earlier risk stratification and targeted diagnostic evaluation.

The retina represents a unique imaging substrate for systemic disease research. As a highly vascularized neural tissue, it reflects systemic microvascular, metabolic, inflammatory, and neurodegenerative processes. Retinal fundus photography, widely used in clinical practice, has been linked to cardiovascular disease, diabetes, renal disease, aging, and mortality, supporting the concept of the retina as a surrogate marker of systemic health [4, 5]. Large-scale imaging cohorts have further enabled quantitative, population-level analysis of these associations [6-8].

Several clinical observations provide biological plausibility for a relationship between CRC and changes in retinal structure. These include cases of intraocular metastases [9, 10], paraneoplastic retinopathies and optic neuropathies [11-13], and systemic effects such as iron-deficiency anemia producing characteristic retinal vascular and nerve fiber layer changes [14] (Fig. **1**). However, these manifestations are rare and not specific to CRC. By contrast, some hereditary colorectal cancer syndromes are specifically characterized by Congenital Hypertrophy of the Retinal Pigment Epithelium (CHRPE) as shown in Table **1** [15-17]. Together, these findings suggest that CRC can influence retinal structure through neoplastic, immune-mediated, vascular, and metabolic mechanisms.

Whether subtle, subclinical retinal signatures specifically associated with CRC exist at the population level remains unclear. Such changes are unlikely to be easily detectable by visual inspection alone but may be present as complex patterns within fundus images. Deep learning (DL) methods are well suited to detecting these patterns, as they can learn high-dimensional image representations without predefined features [18-20]. The present work is complementary to such tissue-based efforts: rather than analysing invasively obtained histopathology, we ask whether a non-invasive retinal image carries CRC-associated signal.

DL applied to retinal fundus photographs has evolved from ophthalmic screening toward systemic risk stratification at population scale. Multiple UK Biobank-based studies have shown that retinal images can be used to predict cardiovascular risk, coronary artery calcification, and future cardiometabolic events, demonstrating incremental prognostic value beyond traditional risk factors [21-24]. Beyond cardiovascular disease, retina-derived models capture broader systemic signals. DL-extracted vascular features and microvascular complexity metrics have been associated with risks of hypertension, heart failure, renal disease, type 2 diabetes, and long-term mortality [25-28]. Aging biomarkers derived from fundus imaging, such as the retinal age gap, have also shown independent associations with survival outcomes [26]. Together, these findings support the premise that retinal photographs encode multidimensional biomarkers of systemic health. Nevertheless, CRC has not been investigated as a primary endpoint in prior UK Biobank retinal DL studies.

Standardized fundus photography databases acquired under controlled conditions have been established to support such image-based analyses [29]. In this context, the UK Biobank provides a valuable resource, offering standardized retinal fundus images linked to health data at population scale. In this study, we investigated whether a DL classifier trained exclusively on retinal fundus photographs from the UK Biobank could distinguish individuals with CRC from those without. Our aim was to assess whether retinal images contain recoverable information associated with CRC status, thereby exploring the structural changes of the retina as a potential imaging-based biomarker of systemic malignancy. To our knowledge, this is the first study to examine CRC status discrimination from retinal fundus photographs at population scale. The intended contribution is therefore the clinical finding rather than a new architecture. We deliberately adopted a standard, well-characterized backbone with transfer learning to prioritize reproducibility and to limit the risk of overfitting through bespoke architectural choices.

## 2 METHODS

### 2.1 Fundus Image Dataset

This study uses retinal fundus photographs from the UK Biobank, a population-scale biomedical resource with comprehensive health, lifestyle, and imaging data from ~500,000 participants [6, 8]. Within this cohort, ophthalmic imaging produced a large archive of color fundus photographs acquired under standardized conditions, enabling epidemiologic analyses of ocular and systemic phenotypes [7, 8, 30]. In line with prior descriptions of the imaging pipeline, photographs were captured on a TOPCON 3D OCT-1000 Mk2 camera with a 45° field of view, centered to include the optic disc and macula, and stored as JPEG (2048×1536 pixels) [8, 30]. At the time of imaging, participants were between 40 and 69 years old, resulting in a collection of 68,000 retinal images acquired between 2009 and 2010 [7].

Data access for the present work was obtained under the approved UK Biobank project ID: 92804. From the available pool, and following manual image quality assessment by a trained expert, two subsets of right eye fundus photographs with satisfactory quality were assembled: (1) CRC-labelled images (CRC, *n* = 800) and (2) a size-matched subset of images from participants without CRC (non-CRC, *n* = 800). Controls were drawn from UK Biobank participants without a CRC record and were count matched to cases in a 1:1 ratio, following the same manual image quality assessment applied to cases. They were not matched on demographic or clinical covariates. This count-matched (rather than confounder matched) design accounts for the residual baseline differences in age and vascular/heart disease observed between the groups, which were instead addressed analytically through a covariates-only baseline model (Statistical Analysis).

CRC status was defined within the approved UK Biobank project context based on available cohort health records. This analysis did not stratify CRC cases by time-from-diagnosis to imaging (*e.g*., prevalent *vs*. incident CRC relative to fundus acquisition) or by treatment status. Consequently, the model may capture correlates of CRC status and/or associated comorbidity or treatment-related signatures; the present study is therefore framed as a feasibility analysis in a curated case-control subset.

This configuration follows prior UK Biobank retinal studies that use standardized acquisition and large-scale curation to investigate disease associations [8, 21, 30, 31]. However, demographic and clinical covariates were not used as model inputs; they were examined only in post-hoc baseline comparisons to assess group comparability and potential confounding.

### 2.2 Retinal Dataset Preparation and Annotation

Preprocessing was deliberately minimal to preserve native appearance and fine anatomical detail. All photographs were converted to grayscale using a luminance transform and scaled per image to the [0, 1] range (pixel values divided by 255). Grayscale conversion was chosen to emphasize structural and morphological retinal features (vascular caliber and branching, neuroretinal texture) over chromatic cues and to reduce sensitivity to inter-acquisition colour and illumination variation, which can act as device- or session-level confounds. The single grayscale channel was later replicated across three inputs solely to retain compatibility with the ImageNet-pretrained RGB stem. Images were resized to 1024×768 (no padding or geometric warping), so that disc-macula geometry and major arteriovenous branches remain faithfully represented [32]. Data augmentation was intentionally simple, horizontal mirroring only, to increase variability while keeping anatomical content intact [33]; given known inter-ocular asymmetries, flipping is treated as a pragmatic variability boost rather than a symmetry assumption [34, 35]. The starting corpus comprised 1,600 images (CRC n=800, non-CRC n=800) and was stratified into 70%/15%/15% training/validation/test splits, yielding 1,120 training images (560 CRC/560 non-CRC), 240 validation images (120/120), and 240 test images (120/120) (Fig. **2A**). The split was performed at the participant level to prevent any subject overlap between training, validation, and test sets (i.e., no participant contributes images to more than one split). Augmentation was applied only to the training subset via horizontal mirroring, increasing the number of training instances presented during optimization without introducing new participants. In addition, the training data loader employed class-balanced sampling with replacement to stabilize optimization and maintain balanced mini-batches throughout training (Fig. **2B**). For clarity, reported split sizes refer to unique images, whereas the effective number of training instances per epoch increases due to mirroring and sampling with replacement (as illustrated in Fig. **2B**). Validation and test splits were never augmented and remained fixed for model selection and final evaluation.

### 2.3 Architecture, Training, and Evaluation Protocol

The study’s experiments framed CRC detection as a binary image-classification problem and relied on transfer learning with an EfficientNet backbone [36, 37]. We selected the B3 variant as a practical middle ground: it offers greater capacity than B0 and can capture fine vascular and macular structure in high-detail fundus photographs, while remaining tractable on a single GPU at high input sizes. The network was initialized with ImageNet-1K weights [38] and fine-tuned end-to-end. Because the fundus images were converted to grayscale during preprocessing, we replicated the single channel three times at the input to maintain compatibility with pretrained RGB filters.

Beyond per-image intensity scaling, we did not apply histogram equalization or additional photometric normalization that could alter subtle vascular contrast, consistent with common transfer-learning practice in medical imaging [39]. Architecturally, we retained the standard EfficientNet-B3 stem and MBConv blocks with squeeze-and-excitation and Swish activation [36, 40, 41], and replaced the classifier with a two-logit output layer trained using cross-entropy loss. Batch-normalization statistics were updated during fine-tuning (no layer freezing), given the domain and resolution shift from ImageNet to fundus photographs [39].

Optimization used AdamW (initial learning rate 1×10^^−4^, weight decay 1×10^^−4^) with a cosine learning-rate schedule and a short warm-up [42, 43]. We added light regularization at the head (dropout *p* = 0.3) and label smoothing (ε = 0.1) to temper overconfidence [44, 45]; stochastic depth was not used to keep the training configuration simple and stable under high-resolution inputs and minimal augmentation [46]. Training proceeded for a maximum of 30 epochs with early stopping on validation loss (patience = 5). Mixed-precision (fp16) and activation checkpointing were enabled to reduce memory usage and increase throughput on large inputs [47, 48]. Runs were executed on an in-house High-Performance Computing (HPC) cluster with NVIDIA L40 GPUs (48 GB); at an input resolution of 1024×768, we used a batch size of 8 and, when needed, gradient accumulation to emulate a larger effective batch while preserving optimizer hyperparameters. Reproducibility was addressed by fixing seeds across Python/NumPy/PyTorch and logging software/hardware metadata; default PyTorch implementations were used [49]. Model selection relied solely on the validation split, and the checkpoint with the lowest validation loss was frozen for evaluation on the held-out test set. For threshold-based metrics, the operating threshold was selected on the validation set (Youden’s index) and applied unchanged to the test set.

### 2.4 Statistical Analysis

Statistical analyses were conducted using Python. Continuous variables are summarized as mean ± SD for interpretability, and categorical variables as counts and percentages. Statistical significance was defined at a two-sided α = 0.05. Model discriminative performance was evaluated using receiver operating characteristic (ROC) analysis, with the area under the ROC curve (AUROC) as the primary metric. For threshold-based metrics (accuracy, macro-F1, sensitivity, specificity, and precision), the operating threshold was selected on the validation set using Youden’s index (sensitivity + specificity − 1) and applied unchanged to the held-out test set. In addition to sample-based diagnostic indices, prevalence-adjusted predictive values (positive/negative predictive value, PPV/NPV) were estimated to approximate performance under population screening conditions. Adjusted PPV and NPV were computed from sensitivity, specificity, and external prevalence assumptions using standard Bayes’ theorem formulations [50].

Baseline demographic and health characteristics were compared between participants with CRC and those without CRC to assess cohort comparability and potential confounding. Normality of continuous variables was assessed using the Shapiro-Wilk test; because several variables deviated from normality, group differences were evaluated using the Mann-Whitney U test. Categorical variables were compared using chi-square tests, with Fisher’s exact test used when expected cell counts were small. To limit over-interpretation from multiple baseline comparisons, *p*-values were adjusted using the Benjamini-Hochberg False Discovery Rate (FDR), and effect sizes are reported alongside significance levels. Effect sizes were quantified using Cliff’s delta (δ) for continuous variables, odds ratios (ORs) for binary variables, and Cramér’s V for categorical variables.

A non-imaging covariates-only baseline model was fitted using age and vascular/heart disease status and evaluated using AUROC to quantify discrimination from these risk factors alone. Baseline-model stability was assessed using stratified 5-fold cross-validation, reporting mean AUROC and standard deviation across folds. No a priori power calculation was performed, because the sample size was fixed by data availability: the study used all CRC-labelled right-eye fundus photographs of satisfactory quality available within the approved UK Biobank extract (n = 800), with non-CRC control count matched 1:1 (total N = 1,600). A formal post-hoc precision and power assessment was therefore performed for the realised design. For the independent test split (120 cases, 120 controls), the Hanley-McNeil standard error of the AUROC corresponds to a 95% confidence interval (CI) half-width of approximately ±0.03 AUROC units at the observed level of discrimination. At a two-sided α = 0.05, this design provides 80% power to reject the null hypothesis of no discrimination (AUROC = 0.5) for a true AUROC of approximately 0.60, and more than 98% power for a true AUROC of 0.65 or greater. The observed discrimination therefore exceeds the minimum detectable effect for this sample size by a wide margin, although the modest absolute size of the held-out splits limits the precision of the point estimates, as reflected in the reported confidence intervals.

### 2.5 Model Explainability and Attribution Mapping (Grad-CAM and Occlusion Sensitivity Analysis)

To examine which retinal regions contributed most strongly to model decisions, we performed post-hoc attribution analyses using two complementary approaches: gradient-weighted class activation mapping (Grad-CAM) and occlusion sensitivity. For Grad-CAM, we extracted activation maps from the final convolutional block of the trained convolutional neural network and combined them with class-specific gradients to obtain a coarse relevance map for the predicted class. The resulting map was upsampled to image resolution and visualized as a heat overlay on the corresponding fundus photograph. Grad-CAM was used primarily for qualitative inspection of broad spatial patterns at a continuous, low-frequency scale. For occlusion sensitivity, we quantified how local perturbations affected the model output. A fixed-size square patch was moved across the image on a regular grid; at each location, the region under the patch was masked (set to a constant baseline), the model was re-evaluated, and the change in the predicted CRC score (probability) was recorded. Larger decreases in score indicate that the masked region contained information that the model relied upon. This procedure produced an occlusion heatmap aligned to the image grid. For visualization and cross-case comparison, occlusion values were min-max normalized to the range [0,^1^]. Cohort-level “average” occlusion maps were obtained by pixel-wise averaging of normalized maps across selected cases (*e.g*., true positives and true negatives). To move beyond qualitative inspection and quantify where attribution concentrates anatomically, we computed the fraction of total occlusion attribution contained within two predefined anatomical regions of interest (ROIs): a macular ROI representing the central retina, and a peripapillary ROI representing the region surrounding the optic disc. ROI definitions were predefined using a simple geometric formulation. The macular ROI was specified as a circular region centred on the posterior pole in the normalized fundus coordinate system, and the peripapillary ROI as an annulus centred on the optic disc. As optic disc segmentation was not performed, the disc center was approximated by a fixed location in the normalised coordinate system and applied uniformly across all images. ROI boundaries were defined computationally in this normalized coordinate system rather than drawn as anatomical contours. Because the optic disc was not segmented and images were not registered to a common coordinate frame, the resulting ROI percentages are anatomically approximate and are reported as descriptive summaries only. Prior to quantification, each occlusion heatmap was restricted to the retinal field using a fundus mask to exclude background pixels outside the circular fundus. Within this retinal mask, we computed the total attribution as the sum of normalized occlusion values across all retinal pixels. We then computed macular and peripapillary attribution as the corresponding sums restricted to each ROI. ROI attribution was reported as a percentage of total retinal attribution (ROI sum divided by the total retinal sum, multiplied by 100). The same ROI definitions were applied consistently across all images, and ROI percentages were summarized separately for the true positive and true negative cohorts.

### 2.6 Covariate-based Comparative Modeling - (Non-Imaging Predictive Model Development)

To evaluate whether baseline risk-factor differences could plausibly account for the observed discrimination, we trained a non-imaging baseline model using only the two variables identified as the most plausible confounders in the baseline comparison: age and a binary indicator of vascular/heart disease (any *vs*. none). No image-derived features were used in this analysis, and the objective was not to maximize overall performance but to quantify the discriminatory capacity of these covariates alone under the same case-control framework used for the image model. Age was treated as a continuous predictor and standardized using z-scoring. Standardization parameters (mean and standard deviation) were estimated on the training data and applied unchanged to the corresponding evaluation data to avoid information leakage. The vascular/heart disease variable was encoded as a binary predictor (0 for absence, 1 for presence of any recorded vascular/heart disease). Records with missing covariate values were excluded from this baseline analysis using a deterministic complete-case approach. The outcome variable matched the primary classification task (CRC *vs*. non-CRC). A logistic regression classifier with L2 regularization was fitted by maximum-likelihood estimation. Logistic regression was selected because it provides an interpretable baseline for assessing the extent to which simple clinical/demographic covariates can separate the two cohorts. Discriminative performance was summarized by AUROC. In addition to reporting AUROC on the balanced held-out evaluation split, we estimated the stability of covariate-only discrimination using stratified 5-fold cross-validation. Within each fold, z-scoring parameters were learned on the training fold and applied to the corresponding validation fold. Cross-validation results were summarized using the mean AUROC and standard deviation across folds.

## 3 RESULTS

### 3.1 Model Performance

ROC analysis on the training split yielded AUROC = 0.904, indicating strong separability within the training data. Importantly, AUROC remained high on the held-out validation and test splits (0.919 and 0.945, respectively; Fig. **3**), suggesting that the learned ranking generalizes to unseen images within the same curation and acquisition setting rather than reflecting a purely training-specific artefact. The ordering of AUROC across splits (training 0.904 < validation 0.919 < test 0.945) is expected rather than anomalous. Training set metrics were computed under the training regime with horizontal-mirroring augmentation, class-balanced sampling with replacement, and active regularization (dropout *p* = 0.3, label smoothing ε = 0.1) in train mode. Validation and test metrics were computed in evaluation mode on un-augmented, un-smoothed images, so the apparent training performance is conservatively biased downward. Given the modest size of the held-out splits (n = 240 each), analytic 95% confidence intervals (Hanley-McNeil) were wide and overlapping (validation 0.919, 95% CI 0.883-0.955; test 0.945, 95% CI 0.915-0.975), indicating that the validation-test difference lies within sampling variability. As summarized in Table **2**, precision, sensitivity (recall), and specificity are all in the low-to-mid 80% range, indicating relatively balanced error profiles without a strong bias toward either class. On the independent test split, sensitivity for CRC was 90.00% (low false-negative (FN) rate), while specificity for non-CRC was 84.17% (moderate false-positive rate). Precision (PPV) for CRC was 85.04%, and macro-F1 was 87.07%, closely tracking the overall accuracy of 87.08%. Threshold-based metrics were computed at the operating point selected on the validation set (Youden’s index) and applied unchanged to the test set.

### 3.2 Prevalence-adjusted Predictive Performance

To estimate real-world translational performance, we applied prevalence-adjusted predictive value formulas using sensitivity and specificity at the Youden index-derived operating threshold. For the UK population aged 40-69 years, colorectal cancer prevalence in 2021 ranged from approximately 0.08%-0.8% [51], depending on the stratified age range. For adjustment of our DL model performance to a UK Biobank-comparable population (ages 40-69), we used a conservative prevalence estimate of 0.5%, taking into account that disease burden increases more in patients ≥60, and the prevalence shows a trend of increase over time. We also modeled performance within a higher-risk subgroup aged 60-69 years, where the reported prevalence was estimated at around 1.2% (Table **3**).

### 3.3 Comparison of Sociodemographic and Clinical Characteristics of the Two Patient Populations

Baseline sociodemographic and clinical characteristics of the CRC and non-CRC cohorts are summarized in Table **4**. Participants with CRC were slightly younger at recruitment than controls (62±6.61 *vs*. 64±3.42 years; *p* < 0.001). Sex distribution did not remain significant after False Discovery Rate (FDR) correction. The groups were otherwise broadly comparable with respect to body mass index, smoking status, alcohol consumption, and physician-diagnosed diabetes. Vascular/heart disease was more prevalent in the non-CRC group than in CRC participants (39.2% *vs*. 32.9%; FDR-adjusted *p* = 0.045). Aside from age and cardiovascular comorbidity, baseline characteristics were comparable between cohorts.

### 3.4 Covariate-only Baseline Model (Risk-factor Confounding Check)

Baseline comparisons (Table **4**) indicated that age and vascular/heart disease were potential confounders. To quantify the discriminatory information contained in these risk factors alone, we trained a covariates-only baseline classifier using age and vascular/heart disease status, without incorporating retinal image information. The model was implemented as a logistic regression with z-scored age and a binary comorbidity indicator and evaluated under the same balanced case-control framework used for the image-based experiments. On the held-out balanced test split, the covariates-only baseline achieved an AUROC of 0.629 (Fig. **4**). Model stability was assessed using stratified 5-fold cross-validation, yielding a mean AUROC of 0.623 ± 0.073 across folds. Overall, these results indicate that age and vascular/heart disease alone provide only modest discrimination and are unlikely to account for the substantially higher performance observed for the image-based model.

### 3.5 Heat Maps for Single Retinal Fundus Image

Figure **5** contrasts a representative non-CRC example with a CRC true-positive example across four complementary views: the original color fundus photograph, an anatomy-focused Grad-CAM overlay, an artefact-focused Grad-CAM overlay, and an occlusion-sensitivity heatmap. Qualitatively, the anatomy-focused Grad-CAM overlay for the true-positive case concentrates on central retinal regions, including the macular area and adjacent segments along major vessels, whereas prominent illumination artefacts receive comparatively lower attribution. Consistently, the occlusion-sensitivity heatmap shows the largest score drop when central regions are masked, with progressively smaller effects toward the periphery, suggesting that the prediction is driven primarily by central retinal regions rather than peripheral areas.

### 3.6 Average Heat Maps for True Positive and True Negative Cohorts

To evaluate whether the patterns observed in a single representative example generalized to the cohort level, we computed average attribution maps across correctly classified cases. For each subject, occlusion sensitivity produced a grid-based heatmap reflecting the magnitude of the model score change under local masking; heatmaps were min-max normalized to [0, 1] and averaged pixel-wise within outcome-defined subgroups. Figure **6** summarizes these cohort-level averages for true positives (TP; CRC cases correctly classified) and true negatives (TN; non-CRC controls correctly classified), alongside the corresponding anatomy-focused and artefact-focused Grad-CAM overlays. In both true positive and true negative cohorts, the averaged occlusion maps indicate that the largest score changes occur in central retinal regions, with comparatively smaller effects toward the periphery. ROI-based quantification supported this pattern: macular attribution accounted for 52.1% of total occlusion attribution in the true positive cohort and 50.9% in the true negative cohort, whereas peripapillary attribution accounted for 9.9% (true positive) and 11.0% (true negative), with the remaining attribution distributed across other retinal regions. Because central emphasis is also consistent with typical fundus image composition, we interpret these cohort-level maps primarily as evidence that the model relies on the central retinal region rather than peripheral or illumination-driven artefacts. These ROI percentages are descriptive summaries and are not intended as inferential statistics. Because the optic disc was not segmented and images were not registered to a common coordinate frame, cohort-averaged maps are expected to emphasize central regions by construction. Accordingly, the near-identical macular percentages in true positives (52.1%) and true negatives (50.9%) should be read as evidence against reliance on peripheral illumination artefacts rather than as anatomically specific, disease-related localization.

## 4 DISCUSSION

This study demonstrates that a deep learning classifier trained exclusively on retinal fundus photographs from the UK Biobank can distinguish individuals with CRC from those without CRC in a class-balanced case-control setting, achieving an AUROC of 0.945, accuracy of 87.08%, macro-F1 of 87.07%, sensitivity of 90.00%, and specificity of 84.17% on a held-out test split. Because the dataset was balanced by design, the close alignment between accuracy and macro-F1 indicates that performance was not driven by a single class. Importantly, discrimination was achieved using retinal images alone - without demographic or clinical covariates - suggesting that, under this curated acquisition and evaluation protocol, image-only models can extract discriminative retinal information correlated with clinically manifest CRC status.

The significance of this finding becomes clear when considered in the context of currently recognized retina-CRC associations. Structural retinal alterations specifically linked to CRC susceptibility are largely confined to hereditary syndromes, most notably CHRPE in FAP, (Table **1**) [15]. Bilateral, multiple (>4 lesions per eye) CHRPE lesions in a characteristic “bear-tracks” configuration demonstrate approximately 95% specificity and 78% sensitivity for Gardner syndrome, a phenotypic variant of FAP [16, 17]. Solitary CHRPE may not be associated with increased risk [52]. Accordingly, the present results are unlikely to be explained by classical FAP-associated CHRPE. In addition, most CRC cases are sporadic (~65%), approximately 25% occur in individuals with a family history, and only ~10% arise from hereditary syndromes [15]. Other reported retinal manifestations - including choroidal metastases, paraneoplastic retinopathy, and anemic retinopathy - are also rare and non-specific, as they can occur in other malignancies [9-14]. In contrast, the present model detected patterns associated with CRC status across cases more broadly, consistent with the possibility that DL captures subtle composite microvascular and neuroretinal signatures that are not immediately apparent by simple visual inspection. Notably, these results reflect discrimination of established disease in a cross-sectional case-control framework rather than early detection of preclinical malignancy.

Adjustment for real-world prevalence substantially altered the predictive value profile of the model. Within the balanced test dataset, PPV was 85.04%, reflecting the artificially elevated prevalence inherent to the case-control design (Table **3**). When recalibrated to a UK population aged 40-69 years using a conservative prevalence estimate of 0.5%, PPV declined to 2.8%. Conversely, NPV increased to 99.94%, indicating a very high probability of true disease absence following a negative result. This means that in a population aged 40-69 years, where the prevalence of colon cancer is 0.5%, testing 600 individuals by using the proposed method could potentially identify 100 individuals with a positive test result, but only about 3 of them would actually have CRC, while the remaining 97 would receive false-positive (FP) results. Conversely, the remaining 500 individuals would test negative, and virtually all of them would truly be CRC-free, consistent with a very high NPV of 99.94%. Therefore, although the proposed test has a very low PPV in this low-prevalence population, its extremely high NPV makes it potentially useful as a triage tool to rule out disease and reduce the need for more invasive diagnostic procedures. When adjusted to a higher-prevalence 60-69 subgroup (1.2%), PPV increased to 6.5%, while NPV remained high (99.86%). These projections are threshold- and prevalence-dependent and should be interpreted as illustrative deployment scenarios rather than fixed operating characteristics. Taken together, they suggest that the principal translational strength of the model - if validated prospectively - may aid in ruling out CRC or enriching a screened population for downstream definitive testing, rather than acting as a stand-alone diagnostic classifier.

Comparison of demographic and health characteristics showed that CRC and non-CRC participants were broadly comparable across examined variables except age and vascular/heart disease. Although the age difference reached statistical significance, the absolute difference was modest (non-CRC *vs*. CRC mean ± SD: 64±3.42 *vs*. 62±6.61). Given that CRC risk does not decrease, but rather increases steeply across broad age strata [51], the observed gradients within narrow intervals are typically smaller and are unlikely, by themselves, to account for the magnitude of discrimination observed. In addition, prior retinal AI work [21] showed that age prediction from fundus photographs becomes reliable primarily across wider temporal margins [21]. In that study, participant age could be predicted from retinal photographs with approximately 79% accuracy only when allowing an error margin of ±5 years, whereas prediction within a narrower ±3-year margin achieved substantially lower accuracy (~54%) [21]. While age-related retinal variation may contribute marginally to classification, it is unlikely to explain model performance in isolation. To further quantify discrimination obtainable from measured risk factors alone, we fitted a covariates-only baseline using age and vascular/heart disease status without image inputs. This model achieved modest discrimination (AUROC 0.629 on the held-out balanced test split; 5-fold cross-validation mean AUROC 0.623 ± 0.073), indicating limited separability when restricted to these variables. While residual confounding cannot be excluded, these results suggest that measured age and vascular/heart disease alone are unlikely to account for the discrimination achieved by the image-only model.

Attribution analyses were broadly consistent with reliance on central retinal content. In cohort-level averages, occlusion-sensitivity maps (score-drop proxies) concentrated predominantly in central regions in both true positive and true negative groups, and ROI quantification indicated that approximately half of total occlusion-based attribution fell within the macular ROI (52.1% in true positives; 50.9% in true negatives), with smaller contributions within the peripapillary ROI (9.9% in true positive; 11.0% in true negative). These findings support spatial stability of model sensitivity at the group level and reduce the likelihood that predictions are driven primarily by peripheral illumination artefacts, although attribution methods cannot establish causality or identify specific biological mechanisms.

CRC has not previously been a primary endpoint in large-scale retinal deep learning studies, limiting direct comparability. However, adjacent literature demonstrates that retina-derived AI biomarkers can predict cardiovascular disease, renal dysfunction, mortality risk, and biological aging, illustrating that DL can extract systemic health information from fundus photographs alone [21-28]. The present findings extend this paradigm into oncology by demonstrating CRC status discrimination using retinal images as the sole input modality.

The study has several methodological strengths, including use of the large and standardized UK Biobank imaging resource [6], a balanced case-control design reducing class bias, minimal preprocessing preserving native retinal structure, and a reproducible EfficientNet-based modeling pipeline. Notably, high discrimination was achieved without incorporation of clinical covariates, enhancing interpretability of image-derived signal capture. The results reinforce the concept of the retina as a multisystem biomarker platform capable of reflecting oncologic as well as cardiometabolic and neurovascular processes. The ability to discriminate CRC status using retinal images alone suggests the presence of systemic microvascular, inflammatory, metabolic, or immune-mediated alterations detectable through AI-based feature extraction. These findings generate a testable hypothesis that some retinal signatures may precede clinical diagnosis, although this possibility requires longitudinal validation.

Beyond the present discrimination task, deep learning has advanced within CRC diagnosis itself, achieving strong performance in the classification of CRC histopathology images and prompting active discussion of whether the technology is ready for routine clinical-practice implementation [53]. Data-driven methods and emerging laboratory markers are also increasingly explored across the wider CRC care continuum, including the perioperative setting: surgical site infection is the most common complication after colorectal surgery and carries substantial morbidity and economic burden, and predictive markers such as butyrylcholinesterase have been associated with its risk and severity [54]. These developments are complementary to the present imaging-based approach and underscore the breadth of data-driven tools now spanning CRC diagnosis, prognosis, and postoperative management.

## 5 STUDY LIMITATIONS

However, important limitations must be acknowledged. Given the cross-sectional design restricted to inference to clinically manifest CRC, these findings should be interpreted as evidence of CRC-associated retinal signatures rather than proof of screening capability or prospective risk prediction. The present study did not account for prior or current CRC treatment exposure, which - through chemotherapy, targeted agents, and immunotherapies - may in some cases alter retinal structure or impair the transparency of clear eye media [55-57]. Consequently, the detected signal may partially reflect these iatrogenic effects rather than the disease status alone. Because this is a class-balanced case-control design, discrimination estimates may be optimistic relative to population screening due to spectrum effects. Therefore, prospective evaluation in representative cohorts is required. As with most deep learning systems, biological interpretability remains limited despite attribution mapping. Use of grayscale images and restrained augmentation may have constrained capture of chromatic or fine textural biomarkers. The experiments were conducted under a fixed analysis protocol using a single random seed; multi-seed sensitivity analysis, a direct RGB versus grayscale comparison, and subject-specific optic disc segmentation with image registration to a common coordinate frame were not performed and are priorities for future work. The attribution analyses are descriptive rather than mechanistic, and residual or unmeasured confounding beyond the measured baseline covariates cannot be excluded. In addition, UK Biobank participants and imaging conditions may not reflect broader clinical populations (selection effects and protocol homogeneity), and generalizability to heterogeneous real-world settings remains untested. Longitudinal cohorts, multimodal integration, and external validation will be required to define temporal relationships, clarify biological mechanisms, and establish clinical utility.

## CONCLUSION

DL analysis of retinal fundus photographs can discriminate individuals with clinically manifest CRC from those without the disease using image data alone. The findings support the presence of CRC-associated retinal signatures potentially reflecting systemic vascular, metabolic, inflammatory, or paraneoplastic processes. Unlike traditional ophthalmic markers largely confined to hereditary syndromes, these signatures may be more subtle and broadly distributed. However, given the cross-sectional design and low population prevalence, the results should not be interpreted as evidence of screening performance. When recalibrated to population prevalence, PPV decreases substantially while NPV remains high, indicating that any potential translational role, if confirmed in prospective studies, would be more consistent with triage/risk-enrichment (rule-out) rather than diagnostic confirmation. A covariates-only baseline using age and vascular/heart disease status achieved only modest discrimination (AUROC ≈ 0.63), supporting that the observed image-based performance is unlikely to be explained primarily by these measured risk factors alone, although residual confounding remains possible. This work extends the retinal changes-as-biomarker paradigm into oncology and motivates longitudinal, multimodal, and external validation studies to assess generalizability, clarify temporal relationships to disease onset, and determine clinical utility in true screening or pre-diagnostic cohorts.

## Figures and Tables

**Fig. (1):**
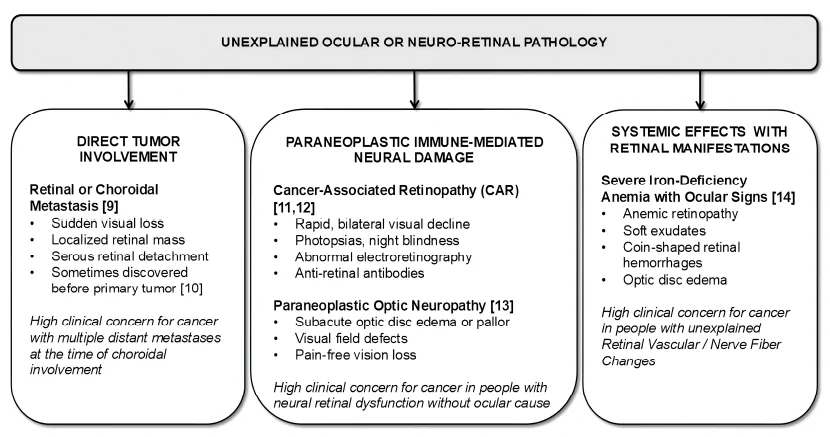
Ocular red flags non-specific to cancer type warranting further oncologic work-up.

**Fig. (2):**
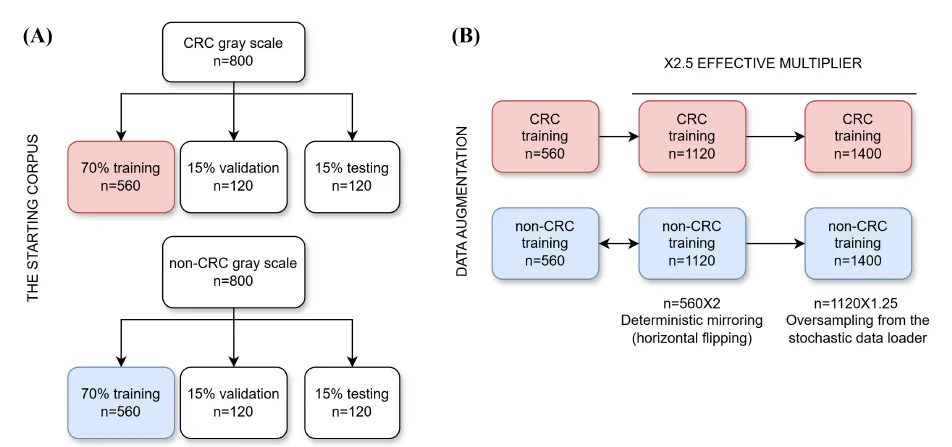
Dataset preprocessing and sample flow. (**A**) Class-balanced corpus converted to grayscale and split into stratified training/validation/test subsets. (**B**) Training-only strategy: horizontal mirroring applied only to the training split; class-balanced sampling with replacement was used within the training data loader to stabilize mini-batches. Validation and test sets were never augmented and remained fixed throughout.

**Fig. (3):**
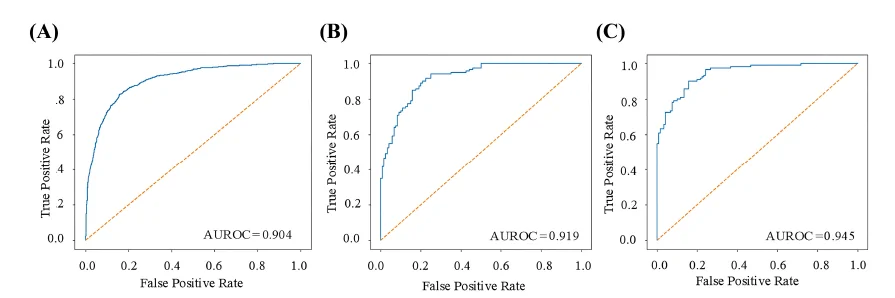
ROC curves (blue line) and values for **A**) Training; **B**) Validation; **C**) Testing.

**Fig. (4):**
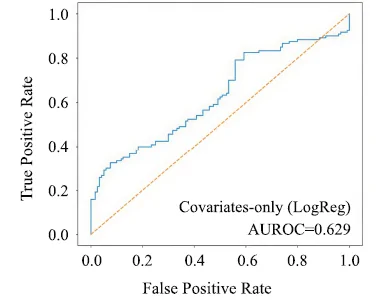
Performance of the covariates-only baseline model using age (as a continuous variable) and vascular/heart disease (any *vs*. none). AUROC on the balanced evaluation split was 0.629.

**Fig. (5):**
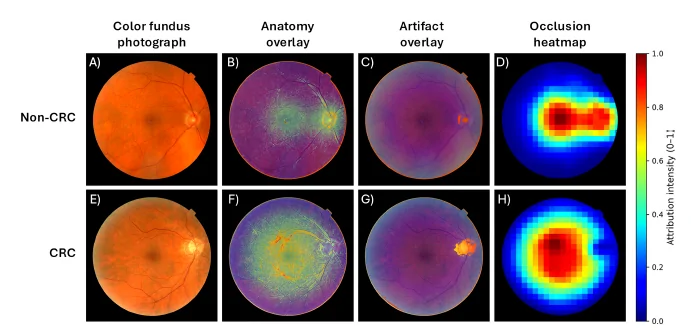
Qualitative comparison of a representative non-CRC (top row) and a CRC true-positive example of a color fundus photograph (bottom row). Original color images are shown for visualization; model inputs were grayscale-converted during preprocessing. Columns show: panels **A**) and **E**) the original color fundus photograph; panels **B**) and **F**) an anatomy-focused Grad-CAM overlay; panels **C**) and **G**) an artefact-focused Grad-CAM overlay; and panels **D**) and **H**) an occlusion-sensitivity heatmap. Color legend: warm colors (yellow-red) indicate higher positive attribution or a larger score drop under occlusion (greater contribution to the decision), whereas cool colors (blue-purple) indicate low contribution. The CRC true-positive case shows concentrated attribution over central retinal regions, while typical illumination artefacts receive comparatively lower attribution. Reproduced by kind permission of UK Biobank^©^.

**Fig. (6):**
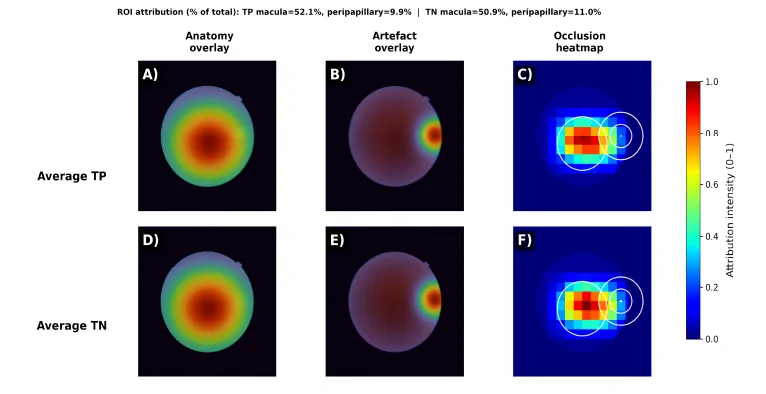
Cohort-level average attribution maps for correctly classified cases. Rows show average maps for true positives (TP; CRC cases correctly classified) and true negatives (TN; non-CRC controls correctly classified). Columns show: **A**) and **D**) - anatomy-focused Grad-CAM overlays, **B**) and **E**) - artefact-focused Grad-CAM overlays (highlighting non-retinal patterns such as illumination gradients and border effects), and **C**) and **F**) - averaged occlusion-sensitivity heatmaps (min-max normalized to [0, 1]) with a common color scale. ROI attribution values report the percentage of total occlusion attribution within macular and peripapillary regions for each cohort.

**Table 1:** Most common high-penetrance genetic syndromes associated with increased risk of CRC.

Syndrome	Primary Gene(s)	Inheritance	Est. Lifetime CRC Risk	Extra-colonic Manifestations	Ocular Manifestations
Lynch Syndrome- Hereditary Nonpolyposis CRC	*MMR* genes: *MLH1, MSH2, MSH6, PMS2*	Dominant	~80%	Endometrial, ovarian, & stomach cancers; sebaceous skin tumors*. *Muir-Torre syndrome is a subtype of Lynch syndrome with skin neoplasms	No.
Classic FAP and Attenuated FAP	*APC*	Dominant	~100% ~70% for attenuated	Desmoid tumors, dental anomalies, thyroid cancer.	Yes- CHRPE-"bear tracks"
Gardner Syndrome- Polyposis with Extracolonic Manifestations [16, 17]	*APC*	Dominant	~100%	Triad: Osteomas (jaw), desmoids, & skin cysts/lipomas.	Yes- CHRPE-"bear tracks"
Turcot Syndrome Type 1 Type2	*MMR* *APC*	Recessive Dominant	High (70-100%)	Brain tumors: Medulloblastoma (with FAP) or Glioma (with Lynch).	Possible (secondary to brain tumor pressure).
MAP	*MUTYH*	Recessive	70-90%	Small bowel polyps; small risk of bladder/ovarian cancer.	No.
Peutz-Jeghers Syndrome	*STK11*	Dominant	~39%	Dark freckles on lips/mucosa; breast & pancreatic cancer.	No.

**Abbreviations:** CRC- colorectal cancer; MMR- mismatch repair; MLH1- MutL Homolog 1; MSH2- MutS Homolog 2; MSH6- MutS Homolog 6; PMS2- Postmeiotic Segregation Increased 2; FAP- Familial Adenomatous Polyposis; APC- Adenomatous Polyposis Coli; CHRPE- Congenital hypertrophy of retinal pigment epithelium; MAP- MUTYH-Associated Polyposis; MUTYH- MutY DNA Glycosylase; STK11- Serine/Threonine Kinase 11.

**Table 2:** Summary metrics for AUROC analysis.

Data Set (n)	AUROC	AP	Accuracy (%)	Macro F1 (%)	Precision (PPV in balanced test set) (%)	Recall (Sensitivity) (%)	Specificity (%)
Training (2800)	0.904	0.901	83.39	83.39	83.95	82.57	84.21
Validation (240)	0.919	0.919	84.58	84.58	84.30	85.00	84.17
Testing (240)	0.945	0.949	87.08	87.07	85.04	90.00	84.17

**Abbreviations:** AUROC-Area Under the Receiver Operating Characteristic; AP- Average Precision; CRC- Colorectal Cancer. Training (n) denotes effective instances per epoch after training-only mirroring and class-balanced sampling with replacement (Fig. **2B**); Validation/test (n) denotes unique images. Threshold-based metrics use the Youden-optimal threshold selected on the validation set and applied unchanged to the test set.

**Table 3:** Prevalence-adjusted performance of the DL model.

Metric	Balanced Test Set	UK Population (age: 40-69)	UK Population (age: 60-69)
Prevalence (%)	50	0.5	1.2
PPV (%)	85.04	2.8	6.5
NPV (%)	89.38	99.94	99.86

**Abbreviations:** UK- United Kingdom; PPV- Positive Predictive Value; NPV- Negative Predictive Value.

**Table 4:** Sociodemographic and clinical characteristics of the two groups of participants.

Variable, Unit of Measurement	Non-CRC	CRC	*p*-value	FDR	Effect
Age at recruitment (years, mean ± SD)	64±3.42	62±6.61	< 0.001*	< 0.001*	*δ*=0.322
Sex (%) Male Female	46.9 53.1	54.0 46.0	0.036	0.087	OR=1.33
BMI (kg/m^2^, mean ± SD)	26.59±4.77	26.87±4.32	0.244	0.292	*δ*=-0.046
Smoking status (%) Never Previous Current Unknown	51.6 40.5 6.5 1.3	50.1 41.5 8.3 0.1	0.066	0.133	*V* =0.083
Alcohol consumption Current drinker Never drinker Previous drinker	89.9 6.5 3.6	92.8 3.8 3.4	0.160	0.240	*V* =0.059
Diabetes (doctor) No Yes Unknown	93.1% 6.5% 0.3%	93.2% 5.9% 1.0%	0.690	0.690	*V* =0.038
Vascular/heart disease (doctor) Any None Unknown	39.2 60.5 0.3	32.9 66.7 0.4	0.015*	0.045*	*V* =0.155

**Abbreviations:** CRC- Colorectal cancer; FDR- False Discovery Rate; BMI- Body Mass Index. FDR indicates Benjamini-Hochberg adjustment for multiple baseline comparisons. Effect sizes: Cliff’s *δ* for continuous variables, odds ratio (OR) for binary variables, and Cramér's *V* for categorical variables. *P*-values < 0.05 are considered significant (labeled with an asterisk).

## Data Availability

This research has been conducted using the UK Biobank Resource under Application Number 92804. UK Biobank data are not publicly available and were used under license for the current study but are available to bona fide researchers through application to the UK Biobank. The code used for data processing, analysis, and model training is available at: https://github.com/ivanjovovic/CRCRetinaAiAGE.

## LIST OF ABBREVIATIONS

AI: Artificial Intelligence
AP: Average Precision
AUROC: Area Under the Receiver Operating Characteristic Curve
BMI: Body Mass Index
CHRPE: Congenital Hypertrophy of the Retinal Pigment Epithelium
CI: Confidence Interval
CNN: Convolutional Neural Network
CRC: Colorectal Cancer
DL: Deep Learning
FAP: Familial Adenomatous Polyposis
FDR: False Discovery Rate
FN: False Negative
FP: False Positive
Grad-CAM: Gradient-weighted Class Activation Mapping
HPC: High-Performance Computing
MAP: MUTYH-Associated Polyposis
MMR: Mismatch Repair
NPV: Negative Predictive Value
OCT: Optical Coherence Tomography
OR: Odds Ratio
PPV: Positive Predictive Value
ROC: Receiver Operating Characteristic
ROI: Region of Interest
SD: Standard Deviation
SSI: Surgical Site Infection
STROBE: Strengthening the Reporting of Observational Studies in Epidemiology
TN: True Negative
TP: True Positive
UK: United Kingdom

## STANDARDS OF REPORTING

STROBE guidelines were followed.
